# Distance measurement between two flexible sites in proteins in high viscosity medium at physiological temperature using continuous wave EPR

**DOI:** 10.1007/s13238-014-0040-5

**Published:** 2014-03-20

**Authors:** Lu Yu, Wei Wang, Shenglong Ling, Yao He, Liang Xiao, Kaiqi Wu, Longhua Zhang, Changlin Tian

**Affiliations:** 1 Hefei National Laboratory for Physical Science at the Microscale & School of Life Science, University of Science and Technology of China, Hefei, 230026 China; 2 High Magnetic Field Laboratory, Chinese Academy of Sciences, Hefei, 230031 China

**Dear Editor,**

Accurate measurement of distances within and between biological macromolecules is important for structure-function studies, for investigating conformational changes and dynamics, and for improving restraints for molecular dynamics and modeling programs (Hellmich and Glaubitz, 2009). Several methods have been developed to measure the distances between atoms, residues, and domains in single proteins and larger complexes. Fluorescence resonance energy transfer (FRET) is currently the most frequently used method to derive distances in cells or protein complexes, but the bulky size of the fluorescence probes and the strict requirement for frequency profile overlap between the acceptor and donor chromophores are limitations in many cases (Prevo and Peterman, 2014). Magnetic resonance of nuclear or electron spins can also provide distance information from the dipolar couplings between two identical spins. However only short inter-atomic distances (<6 Å between two protons) can be measured using this approach. In contrast, electron paramagnetic resonance (EPR) dipolar coupling between two electrons can be applied for distance measurements up to 80 Å (Lakshmi and Brudvig, 2001), provided the proteins coordinating the two unpaired electrons are relatively rigid (i.e. minimal motion; Altenbach et al., 2001).

In protein EPR studies, unpaired electrons can be introduced through site-directed spin labeling of methanethiosulfonate (MTSL, R1) at cysteine residues through disulfide bridge formation between MTSL and cysteine. EPR signals acquired from the introduced R1 groups can then be quantified for determination of mobility and accessibility (Sompornpisut et al., 2008). Upon introduction of two R1 sites, distances from 8 Å to 20 Å can be extracted using continuous wave (CW) EPR spectroscopy via line-shape analysis (Banham et al., 2008), while distances between 20 Å and 80 Å can be obtained using double electron-electron resonance (DEER) pulsed EPR methods (Li et al., 2012; Sale et al., 2005; Wu et al., 2013). Conditions of “rigid-limit” are similarly required for both methods.

In theory, quantitative analysis of inter-spin distances must be carried out in the absence of dynamic effects, which is normally achieved by freezing the sample. Under rigid-limit conditions (<200 K), elimination of the residual motions of both R1 groups allowed the precise dipolar interaction between two approximate spins to be calculated (Hustedt and Beth, 1999). Unfortunately, such low temperatures may restrict the dynamics to the extent that important functional information is lost. If the molecular tumbling rate was slow relative to the magnitude of the dipolar coupling, dipolar broadening would not be averaged, and the broadening function could then be measured at room temperature, where the protein rotational correlation time meets the condition τ_c_ ⩾(r^3^h)/(3πg^2^β^2^) (where r is the inter-spin distance, h is Planck’s constant, g is the g factor, and β is the Bohr magneton) (Altenbach et al., 2001). Elevating the viscosity represents an alternative way to decrease the protein rotational correlation time (τ_c_) in order to achieve accurate inter-spin distance measurements at physiological temperatures. Previously, MTSL labeling at two sites in a rigid helix allowed accurate distance measurement at physiological temperature, and the rotational correlation time was decreased by addition of sucrose (Altenbach et al., 2001). However, spin radical labeling at two flexible sites located in different secondary structure elements has not been reported yet.

In order to investigate the effect of viscosity on distance measurements between two flexible sites, MTSL labeling was performed on T4 lysozyme (T4L). Singly labeled S90R1, singly labeled S117R1, and doubly labeled S90R1/S117R1 were prepared. Glycerol was included in the aqueous samples at 0, 15, 50 and 80% (*w*/*w*) concentrations to generate different viscosities at physiological temperature. From the crystal structure of T4L (PDB code 2LZM), residue 90 is in a loop region exposed to solvent, while residue 117 is found in a helix boundary facing the interior of the protein (Fig. 1A). The two sites (90 and 117) are in close proximity (1.32 nm between C_β_-C_β_ and 1.38 nm between C_α_-C_α_), within the range of interactions that can theoretically be measured using CW-EPR.

**Figure 1:**
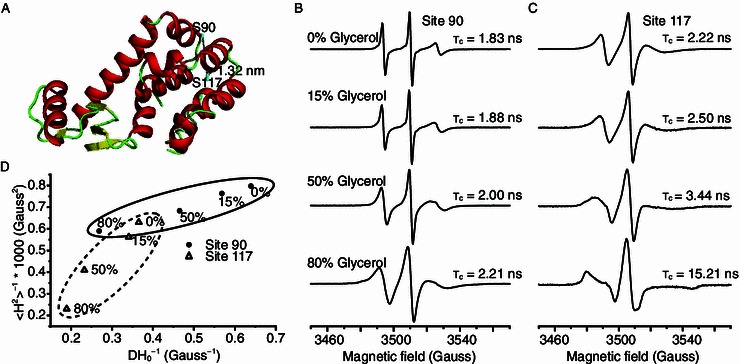
**Mobility analysis of T4L-S90R1 and T4L-S117R1 T4 lysozyme using CW-EPR**. (A) Spin radicals were labeled at two sites of T4L. Site 90 is part of a flexible loop, while site 117 is part of a helix that is buried in the protein core. (B) EPR spectra of T4L-S90R1 and (C) T4L-S117R1 at 298 K, in aqueous buffer containing different glycerol concentrations, obtained with a magnetic field scan width of 200 G. Rotational correlation times are shown below each spectrum. (D) Correlation of the mobility parameters 〈H^2^〉^−1^ (inverse second moment) and ΔH_0_^−1^ (inverse of the central line width) for S90R1 and S117R1. Mobility parameters were calculated from the CW-EPR spectra shown in (B) and (C). Ovals indicate the clustering effect

The mobility of the spin labeled T4L samples in buffers containing different glycerol concentrations were subjected to CW-EPR spectroscopy at room temperature (298 K). T4L-S90C and T4L-S117C proteins were over-expressed, purified and radical labeled as described in the supplementary material (Fig. S1). The mobility of side chain R1 was studied for T4L-S90R1 and T4L-S117R1. The line width of S90R1 undergoes apparent broadening when the glycerol concentration is increased from 0% to 80%, indicating that the motion is gradually attenuated with increasing viscosity (Fig. 1B). For S117R1 (Fig. 1C), a similar pattern was observed, and the spectrum for the sample containing 80% glycerol was typically of a heavily immobilized protein with highly restricted motion of R1. The calculated τ_c_ values of the two singly labeled mutants similarly increased with increasing glycerol concentration and hence viscosity (Fig. 1B and 1C). The rotational motion of S117R1 was slower than that of S90R1, as indicated by the larger correlation time and broader line width for high and low field peaks from solutions of equal viscosity (Essen et al., 1998). This is consistent with the locations of the two labeled sites: residue 90 being solvent-exposed on a surface loop, and residue 117 being buried in the protein core (Fig. 1A).

To further investigate the influence of viscosity on the dynamic features of the two R1 sites, two semi-empirical parameters, 〈H^2^〉^−1^ and ∆H_0_^−1^, were derived from the spectra to extract motional information (McHaourab et al., 1996). The plot of 〈H^2^〉^−1^ versus ∆H_0_^−1^ reflects the degree of motional restriction, with the second moment 〈H^2^〉 related to the averaging of the hyperfine anisotropy, while the central line width, ∆H_0_ is related to the averaging of the g-tensor. The mobility of S90R1 and S117R1 rapidly decreased with increasing viscosity (Fig. 1D), which suggests that high viscosity approaches the rigid-limit conditions required for this type of analysis.

To exclude the possibility that high glycerol concentrations were disrupting the protein structure, a CW-EPR power saturation experiment and accessibility analysis was carried out for T4L-S90R1 and T4L-S117R1 in the presence of 15, 50 and 80% glycerol. The saturation behavior of T4L-S90R1 and T4L-S117R1 was characterized to analyze the local environment of the spin labels, which could be modified by addition of polar relaxants (NiEDDA) with a preference for the aqueous phase, or by nonpolar reagents (O_2_) with a preference for the hydrophobic region (Fig. S2). T4L-S90R1 showed a higher accessibility to polar relaxants than T4L-S117R1 (Fig. S2A–C), indicating a greater degree of exposure for S90R1, as would be expected of a residue belonging to a solvent-exposed surface loop residing in hydrophilic environment. Both T4L-S90R1 and T4L-S117R1 showed a similar but decreased accessibility to O_2_ (Fig. S2D–F), indicating that the motions of the two sites was restricted in the presence of higher glycerol concentrations. These results strongly suggest that no secondary structural changes took place when glycerol was added. This is important to allow the accurate measurement of distances in intact, correctly folded proteins.

Distance measurement was also conducted between S90R1 and S117R1 in different viscosities. In theory, short distances (8–20 Å, or 0.8–2 nm) can be extracted from EPR spectra under rigid-limit conditions from line-shape analysis using convolution or deconvolution methods (Rabenstein and Shin, 1995). To obtain the dipolar broadening effect or inter-spin distances, spectra for the S90R1 and S117R1 singly-labeled mutants and the S90R1-S117R1 doubly-labeled mutant were acquired at physiological temperature with increasing concentrations of glycerol (Fig. 2A–D). The average distance values were derived as 1.82 nm, 1.86 nm, 1.72 nm and 1.62 nm in 0, 15%, 50% and 80% glycerol, respectively (Fig. 2A–D). As a reference, rigid-limit spectra were also acquired in 30% glycerol (as cryo-protectant) at 150 K, and the inter-spin distance was calculated as 1.56 nm (Fig. 2E). From the descent tendency of calculated distance values of T4L at physiological temperature, the approaching to the “rigid-limit” condition (1.56 nm at 150 K) could be observed with the increased viscosity. Apparently, high medium viscosity could restrain the spin mobility and the global molecular motion, leading to the required effects similar as “rigid-limit” condition.

**Figure 2:**
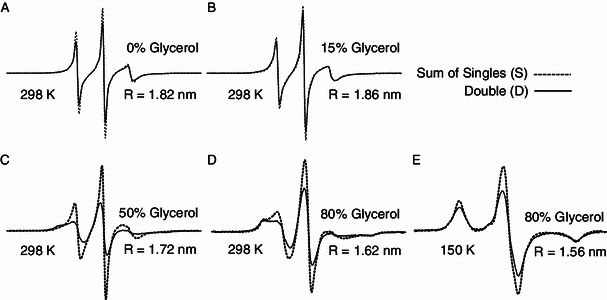
**Distance measurements between two labelled spin radicals in T4 lysozyme**. EPR spectra of S90R1 and S117R1 of T4L with different concentrations of glycerol at physiological temperature (298 K, A, B, C, D) and at low temperature (150 K, E). The light dotted line shows the sum of the singly-labeled sample spectra. The thick line shows the spectrum of the doubly-labeled sample normalized to the same number of spins as the corresponding summed singly-labeled spectra. All spectra were recorded using a 200-G scan width. The calculated distances are shown

In order to achieve a reliable distance measurement, the rotational correlation time should be τ_c_ ⩾(r^3^h)/(3πg^2^β^2^) (Altenbach et al., 2001), which gives the effective τ_c_ required for a specific distance r. Previously, a viscosity of 0.03 P (30% sucrose or 40% glycerol) at room temperature was reported to be sufficient for this purpose, but the spin radical R1 was introduced within a rigid helix (Mchaourab et al., 1996). For R1 labeling of loop regions or other sites with high flexibility, motional correlation time estimation from CW-EPR spectra is required to verify the accuracy of the measured distance. Based on the above equation, for spins separated in by 7–8 Å, both conditions of τ_c_ > 2 ns and τ_c_ > molecular correlation time (here ~6 ns for T4 lysozyme in aqueous buffer at physiological temperature) are required to avoid averaging the major dipolar interaction. For the longer distance of ~16 Å in this study (S90R1–S117R1 in T4L), a τ_c_ > 15 ns is sufficient. The rotational correlation time τ_c_ of either site was below 10 ns for samples containing less than 80% glycerol (Fig. 1B and 1C), while the τ_c_ of T4L-S117R1 reached 15.21 ns in 80% glycerol. Therefore, with rotational correlation time of the either site approaching the required τ_c_ (15 ns for 16 Å), the measured distance (Fig. 2A–E) using the CW-EPR method was getting close to the distance of the two spin sites in real “rigid-limit” (Fig. 2E).

In summary, we have shown that increasing the viscosity of a protein solution using glycerol can successfully mimic the rigid limit conditions of the frozen state that are required to allow the accurate measurement of distances between two flexible radical labeled sites (S90R1 and S117R1 in T4L) using CW-EPR at physiological temperature. However, careful estimation of the rotational correlation time is required to achieve precise and reliable distance measurements using this approach.

## Electronic supplementary material

Below is the link to the electronic supplementary material.

Supplementary material 1 (PDF 215 kb)

## References

[CR1] Altenbach C, Oh KJ, Trabanino RJ, Hideg K, Hubbell WL (2001). Estimation of inter-residue distances in spin labeled proteins at physiological temperatures: experimental strategies and practical limitations. Biochemistry.

[CR2] Banham JE, Baker CM, Ceola S, Day IJ, Grant GH, Groenen EJ, Rodgers CT, Jeschke G, Timmel CR (2008). Distance measurements in the borderline region of applicability of CW-EPR and DEER: a model study on a homologous series of spin-labelled peptides. J Magn Reson.

[CR3] Essen LO, Siegert R, Lehmann WD, Oesterhelt D (1998). Lipid patches in membrane protein oligomers: crystal structure of the bacteriorhodopsin–lipid complex. Proc Natl Acad Sci USA.

[CR4] Hellmich UA, Glaubitz C (2009). NMR and EPR studies of membrane transporters. Biol Chem.

[CR5] Hustedt EJ, Beth AH (1999). Nitroxide spin–spin interactions: applications to protein structure and dynamics. Annu Rev Biophys Biomol Struct.

[CR6] Lakshmi KV, Brudvig GW (2001). Pulsed electron paramagnetic resonance methods for macromolecular structure determination. Curr Opin Struct Biol.

[CR7] Li J, Shi C, Gao Y, Wu K, Shi P, Lai C, Chen L, Wu F, Tian C (2012). Structural studies of *Mycobacterium tuberculosis* Rv0899 reveal a monomeric membrane-anchoring protein with two separate domains. J Mol Biol.

[CR8] McHaourab HS, Lietzow MA, Hideg K, Hubbell WL (1996). Motion of spin-labeled side chains in T4 lysozyme. Correlation with protein structure and dynamics. Biochemistry.

[CR9] Prevo B, Peterman EJ (2014). Forster resonance energy transfer and kinesin motor proteins. Chem Soc Rev.

[CR10] Rabenstein MD, Shin YK (1995). Determination of the distance between two spin labels attached to a macromolecule. Proc Natl Acad Sci USA.

[CR11] Sale K, Song L, Liu YS, Perozo E, Fajer P (2005). Explicit treatment of spin labels in modeling of distance constraints from dipolar EPR and DEER. J Am Chem Soc.

[CR12] Sompornpisut P, Roux B, Perozo E (2008). Structural refinement of membrane proteins by restrained molecular dynamics and solvent accessibility data. Biophys J.

[CR13] Wu K, Shi C, Li J, Wang H, Shi P, Chen L, Wu F, Xiong Y, Tian C (2013). Efficient long-distance NMR-PRE and EPR-DEER restraints for two-domain protein structure determination. Protein Cell.

