# Supplementary material for: Distance measurement between two flexible sites in proteins in high viscosity medium at physiological temperature using continuous wave EPR
Source: Protein Cell. 2014 Mar 20;5(5):334–7. doi: 10.1007/s13238-014-0040-5 (PMC3996158; doi:10.1007/s13238-014-0040-5)
Supplement: Supplementary file 1 — Supplementary material 1 (PDF 215 kb) [file 13238_2014_40_MOESM1_ESM.pdf]

Distance Measurement between Two Flexible Sites of Proteins in High Viscosity Medium at Physiological Temperature using Continue Wave Electron Paramagnetic Resonance

Lu Yu[1], Wei Wang[1], Shenglong Ling[1], Yao He[1], Liang Xiao[1], Kaiqi Wu[1], Longhua Zhang[1], Changlin Tian [1,2,*]

[1] Hefei National Laboratory for Physical Science at the Microscale & School of Life Science, University of Science and Technology of China, Hefei, Anhui 230026, P. R. China
[2] High Magnetic Field Laboratory, Chinese Academy of Sciences, Hefei, Anhui 230031, P. R. China

1. Construction of Cysteine Mutations in T4 Lysozyme.

Mutants of T4L will be designated by giving the single letter code for the original residue, the sequence number, and the single letter code for the new residue. For example, in the T4L mutant S90C, the native Ser residue at position 90 is replaced with Cys. Mutants with more than one substitution are identified by specifying each single mutation separated by a slash. The cysteine-free "pseudo" wild type lysozyme gene containing the mutations C54T and C97A was synthesized (Sangon, Shanghai) and used as template for mutagenesis. Two single cysteine mutants S90C, S117C and one double cysteine mutant S90C-S117C were generated by site-directed mutagenesis using the PCR-based overlap extension method. The mutations were introduced into the plasmid expression vector pET21b, which carries a C-terminal His-tag configuration. Sequencing of the whole lysozyme gene was carried out to verify the mutations.

2. Protein Expression and Purification

Successfully mutated vectors were transferred into *E. coli* BL21(DE3) cells, and colonies were grown overnight at 37 °C on LB/agar/ampicillin plates. Mutants were expressed at 37 °C in LB broth (OXOID) containing 100 μg/ml ampicillin, inoculated with cell stock solutions from single colonies incubated overnight in LB medium at 37 °C. The protein expression was induced by adding Isopropyl-β-D-thio-galactoside (IPTG) to a concentration of 0.8 mM to the cell culture at an OD of 0.8. Cells were harvested by centrifugation at 4000 rpm for 20 min at 4 °C within 4 hours after the induction and resuspended in 40 mL of lysis buffer [70 mM Tris–HCl, 300 mM NaCl, pH 8.0]. Cell suspensions in lysis buffer were probe-sonicated (VC500, Sonics and Materials, Danbury, CT) at a power level of 30%, 2.0 s pulse on and 4.0 s pulse off, for a total of 10 min on ice. The lysate was then centrifuged at 16,000 rpm for 20 min at 4 °C. Pellets from the centrifugation were discarded and the supernatant was mixed with 5 mL of Ni^{2+}-NTA resin (QIAgen, Valencia, CA). The mixture was rotated at

4 °C for 20 min before packing onto a gravity-flow column. Impurities were washed out by applying 50 mL of washing buffer [20 mM Tris, 200 mM NaCl, 20 mM imidazole, pH 8.0] to the column. The target proteins were then eluted from the column using elution buffer [20 mM Tris, 200 mM NaCl, 250 mM imidazole, pH 8.0]. Protein concentration was determined by UV absorption at 280 nm using an extinction coefficient of 1.228 cm2 mg-1. All mutants were at least 95% pure analyzed using SDS-PAGE. For storage DTT was added to a concentration of ~10 mM and the samples were frozen at from −20 to −80°C.

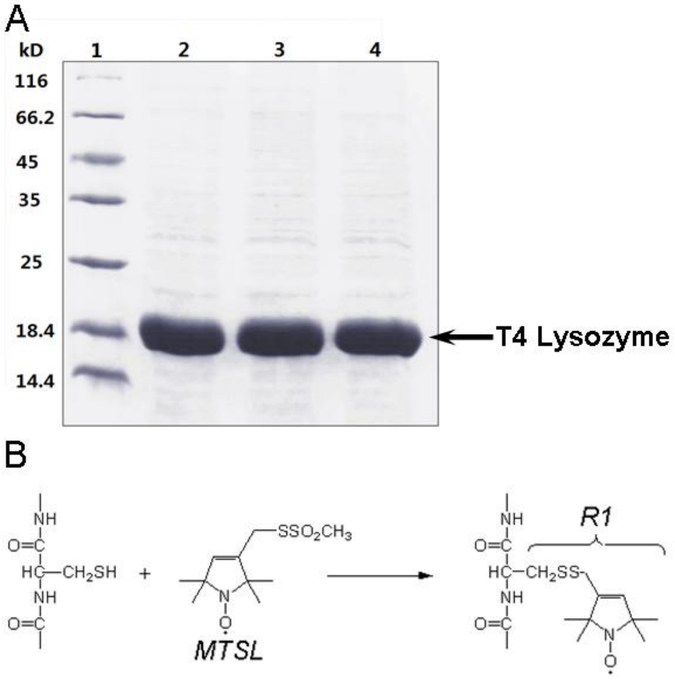

Figure S1. T4 lysozyme purification and site specific spin labeling. (A) SDS-PAGE of purified T4L. Lane 1: molecular weight protein markers; Lane 2: cysless T4 lysozyme (with two mutations C54T and C97A); Lane 3: T4 lysozyme with S90C; Lane 4: T4 lysozyme with S117C. (B) Scheme of site specific spin radical MTSL labeling through disulfide bond at a Cysteine site.

3. Site Specific Spin Radical MTSL Labeling

Prior to spin labeling, DTT was removed using a PD-10 gravity flow desalting column (GE Biosciences), eluting with binding buffer(20 mM Tris , 200 mM NaCl , pH 8.0). Single or double cysteine mutants were immediately reacted with a 10-fold or 20-fold molar excess of MTSL (1-oxyl-2,2,5,5-tetramethyl-Δ3-pyrr-oline-3-methyl methanethiosulfonate, Toronto Research Chemicals, Ontario, Canada) spin label at room temperature for 30 min and then at 4°C overnight. Excess spin reagent was removed using the above mentioned desalting column, eluting with binding buffer. Spin-labeled protein solutions were concentrated to ~200 μM using an Amicon Ultra 15 mL device (10,000 MWCO, Millipore). For samples under different glycerol concentrations, glycerol was added to the spin labeled lysozyme to final concentrations of 0%, 15%, 50% and 80% by weight. Labeling efficiency was determined by measuring the protein concentration and the spin concentration. As determined by double integration of the EPR spectra, the labeling efficiency for each

mutant was over 90%, which was necessary to observe the dipolar interactions as a broadening in the line width of the EPR spectrum of a doubly labeled sample, as compared to a singly labeled molecule.

4. Continue Wave EPR Spectroscopy and Correlation Time Simulation

Solution CW EPR spectra were collected at X-band (9.5 GHz) using a Bruker A300 spectrometer (Bruker Biospin GmbH, Rheinstetten, Germany) equipped with a high-sensitivity cavity (ER 4119HS, Bruker Biospin GmbH, Rheinstetten, Germany) at room temperature (298 K). Spectra were recorded at a microwave power of 2 mW over a scan width of 200 G with a field modulation of 1 G at a frequency of 100 kHz. Samples were placed in a glass capillary tube and had a volume of ~20 μL. A finger dewar insert and a Bruker variable temperature control unit were used to conduct the measurement at low temperature (150K) to eliminate motional averaging of dipolar interactions. A microwave power of 0.24 mW was used to avoid signal saturation at low temperature.

Rotational correlation time (τ_c) of T4L-S90R1 and T4L-S117R1 with different glycerol concentrations was estimated (S. A. Goldman 1972) (Oppenheim et al., 1996)). Briefly, EPR spectra of the labeled protein in solution and at its rigid limit (frozen solution at 150 K), are required, τ_c is evaluated from the expression,

$$\tau_c = a(1-S)^b \qquad (1)$$

where

$$S = A'_{zz}(G) / A^R_{zz}(G) \qquad (2)$$

A^R_{zz} is identical to the magnetic tensor A_{zz} and A'_{zz} is the generic room-temperature genera magnetic tensor. The values of $a = 8.52 * 10^{-10} s$ and $b = -1.16 * 10^{-10} s$ are determined by evaluating the peak-to-peak derivative Lorentzian linewidths (δ) derived from the rigid limit EPR spectrum.

5. CW EPR Power Saturation Studies and Accessibility Analysis

Power saturation experiments were performed on the same spectrometer coupled with an ER 4123D CW resonator (Bruker BioSpin). Samples were loaded into gas permeable TPX capillary tubes with a total volume of 3−4 μL at a concentration of 30−50 μM. EPR data were collected using a modulation amplitude of 1 G and a scan range of 15 G. The range of the incident microwave power was 0.7 to 45 mW for power saturation experiments. Nitrogen is used as a control to purge the sample of oxygen and other paramagnetic relaxing agents. The water-soluble paramagnetic reagent, nickel(II)-EDDA complex (NiEDDA) was synthesized as previously

described (Altenbach et al., 1994). The power saturation curves were obtained for the S90C and S117C under three conditions: (1) equilibrated with lipid-soluble paramagnetic reagent 20% oxygen (air), (2) equilibrated with nitrogen as a control, and (3) equilibrated with nitrogen in the presence of NiEDDA (5 mM).

Power saturation curves were measured as the vertical peak-to-peak amplitude (A) of the first derivative $M_{i=0}$ line as a function of incident microwave power (P). The data points were then fit using an R software script according to eq 3:

$$A = I\sqrt{P}[1+(2^{1/\varepsilon}-1)P/P_{1/2}]^{-\varepsilon} \quad (3)$$

where I is a scaling factor, $P_{1/2}$ is the power where the first derivative amplitude is reduced to half of its unsaturated value, and ε is a measure of the homogeneity of saturation of the resonance line. The change in $P_{1/2}$, $\Delta P_{1/2}$, is calculated as the difference in $P_{1/2}$ values in the presence and absence of relaxing agent. The parameter $\Delta P_{1/2}/\Delta H_{pp}$ is normalized to the same parameter for a reference sample to account for instrumental variations, where ΔH_{pp} is the peak-to-peak linewidth of the first derivative spectrum. The corresponding accessibility parameter, Π, is calculated by the following equation:

$$\Pi = \{\Delta P_{1/2}/\Delta H_{pp}\}/\{P_{1/2}(\mathrm{DPPH})/\Delta H_{pp}(DPPH)\} \quad (4)$$

where $P_{1/2}$(DPPH) and ΔHpp (DPPH) are the $P_{1/2}$ and line-width values determined for a standard sample of crystalline 2,2-diphenyl-1-picrylhydrazyl (DPPH) in KCl, respectively.

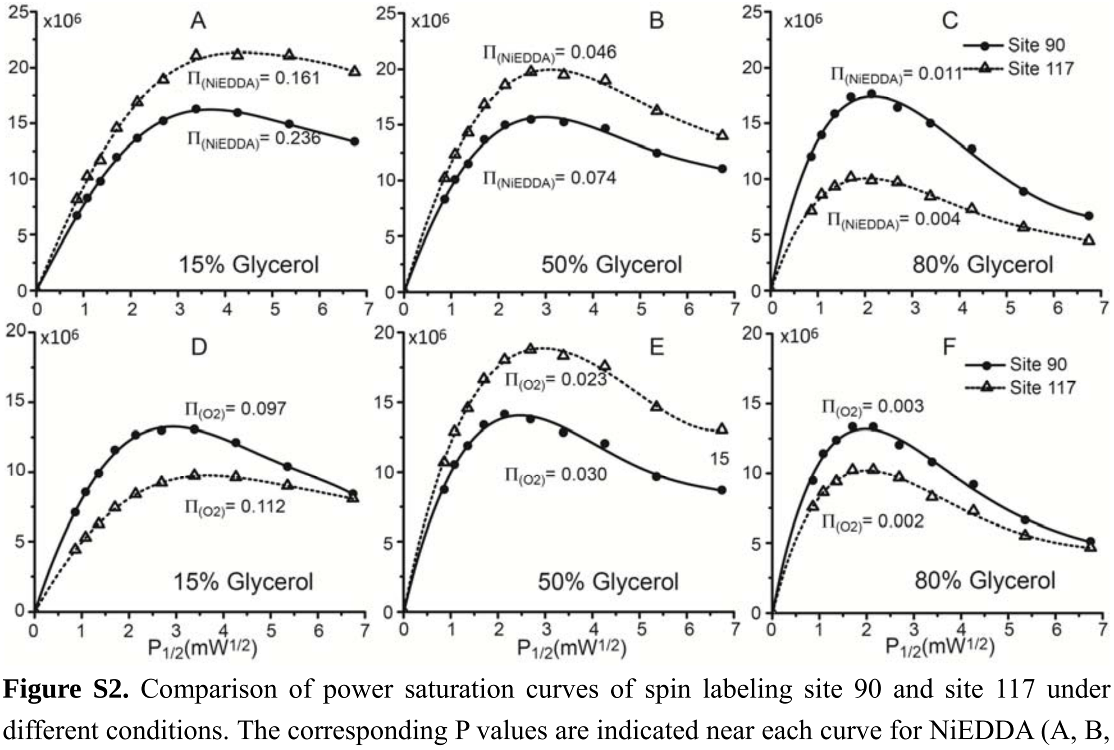

Figure S2. Comparison of power saturation curves of spin labeling site 90 and site 117 under different conditions. The corresponding P values are indicated near each curve for NiEDDA (A, B, C) and for O_2 (D, E, F)

Power saturation CW-EPR is the most common EPR technique to obtain information on side chain accessibility at room temperature, which consists of detecting spectra at increasing microwave power to induce saturation (Altenbach et al., 2005). The amplitude of the central line is plotted against the square root of the incident microwave power, and the saturation curve is analyzed to obtain the empirical $P_{1/2}$ values (the power at which the intensity of the line is half of what it should be in the absence of saturation) and subsequently the Π values. The saturation behavior is characteristic of the local environment of the spin label and can be modified by addition of polar relaxants (neutral NiEDDA) with preference for the aqueous phase or nonpolar reagent (O_2) with preference for hydrophobic core. The difference in $P_{1/2}$ (or Π) in the presence and absence of paramagnetic species indicated the relative collision frequency, or the relative "accessibility" of the spin labels towards the exchange reagent. Thus, a greater value of Π in NiEDDA for a specific site stands for higher accessibility towards hydrophilic environment while a greater value of Π in O_2 represent that this site is more buried in protein core. As shown in Figure S2, site 90 had higher values of Π in NiEDDA than site 117 (Fig. S2A, S2B, S2C), resulting from more exposure to aqueous environment of site 90. Both sites showed resemblance of accessibility under oxygen ambience (Fig. S2D, S2E, S2F). However, as the glycerol concentration increased, the tumbling of the protein is more restricted so the intrinsic features of each site began to reveal. In Fig. S2E and Fig. S2F, the Π value in O_2 at site 117 was observed higher than that at site 90. For both sites, Π values decreased significantly with increasing glycerol concentration. The trend is much more remarkable for Π values in NiEDDA than those in O_2, suggesting that power saturation CW EPR in polar relaxants is more reliable for soluble proteins.

6. Distance Derivation from Dipolar Coupling between Two Spins

Continuous wave EPR method can be used to measure distance or distance distributions (8 ~ 20 A) between two spins at low temperature or room temperature. Spectral broadening of the doubly labeled samples, compared with the singly labeled sample representing essentially no dipolar interaction, was analyzed using Monte Carlo/Simplex Gaussian convolution method ((Rabenstein and Shin, 1995) (Steinhoff et al., 1997)) using a software developed in Dr. Piotr Fajer's laboratory (Fajer et al., 2007).

Briefly, the EPR spectrum can be treated as a convolution of the sum of the spectra of R1 at the individual sites with an appropriate broadening function to represent the dipolar interaction (Rabenstein and Shin, 1995). Such spectral broadening due to spin-spin interaction is readily detected by an amplitude decrease (and line width increase) in the EPR spectrum of the doubly labeled mutant with respect to the sum of the EPR spectra of the single mutants.

References:

Altenbach, C., Froncisz, W., Hemker, R., McHaourab, H., and Hubbell, W.L. (2005). Accessibility of nitroxide side chains: absolute Heisenberg exchange rates from power saturation EPR. Biophys J 89, 2103-2112.

Altenbach, C., Greenhalgh, D.A., Khorana, H.G., and Hubbell, W.L. (1994). A collision gradient method to determine the immersion depth of nitroxides in lipid bilayers: application to spin-labeled mutants of bacteriorhodopsin. Proc Natl Acad Sci U S A 91, 1667-1671.

Fajer, P.G., Brown, L., and Song, L. (2007). Practical pulsed dipolar ESR (DEER). ESR Spectroscopy in Membrane Biophysics (Hemminga,M & Berliner, L, eds) 27, 95-128.

Oppenheim, S.F., Buettner, G.R., and Rodgers, V.G.J. (1996). Relationship of rotational correlation time from EPR spectroscopy and protein-membrane interaction. J Membrane Sci 118, 133-139.

Rabenstein, M.D., and Shin, Y.K. (1995). Determination of the distance between two spin labels attached to a macromolecule. Proceedings of the National Academy of Sciences of the United States of America 92, 8239-8243.

S. A. Goldman , G.V.B., J. H. Freed (1972). Estimating slow-motional rotational correlation times for nitroxides by electron spin resonance.

Steinhoff, H.J., Radzwill, N., Thevis, W., Lenz, V., Brandenburg, D., Antson, A., Dodson, G., and Wollmer, A. (1997). Determination of interspin distances between spin labels attached to insulin: comparison of electron paramagnetic resonance data with the X-ray structure. Biophysical journal 73, 3287-3298.
